# Xuezhikang, an extract from red yeast rice, attenuates vulnerable plaque progression by suppressing endoplasmic reticulum stress-mediated apoptosis and inflammation

**DOI:** 10.1371/journal.pone.0188841

**Published:** 2017-11-30

**Authors:** Linghong Shen, Zhe Sun, Shichun Chu, Zhaohua Cai, Peng Nie, Caizhe Wu, Ruosen Yuan, Liuhua Hu, Ben He

**Affiliations:** 1 Department of Cardiology, Shanghai Chest Hospital, Shanghai Jiaotong University School of Medicine, Shanghai, China; 2 Department of Cardiology, Renji Hospital, Shanghai Jiaotong University School of Medicine, Shanghai, China; Duke University School of Medicine, UNITED STATES

## Abstract

Xuezhikang (XZK), an extract of red yeast rice, is a traditional Chinese medicine widely used for the treatment of cardiovascular diseases in China and other countries. However, whether XZK treatment can improve atherosclerotic plaque stability is not fully understood. Based on our previously developed mouse model of spontaneous vulnerable plaque formation and rupture in carotid arteries in ApoE^-/-^ mice. We showed that low-dose (600 mg/kg/d) XZK improved plaque stability without decreasing plaque area, whereas high-dose (1200 mg/kg/d) XZK dramatically inhibited vulnerable plaque progression accompanied by decreased plaque area. Mechanistically, XZK significantly suppressed lesional endoplasmic reticulum (ER) stress in mouse carotid arteries. *In vitro*, XZK inhibited 7-KC-induced activation of ER stress in RAW264.7 macrophages, as assessed by the reduced levels of p-PERK, p-IRE1α, p-eIF2α, c-ATF6, s-XBP1, and CHOP. Compared to controls, the XZK-treated group displayed dramatically decreased apoptotic cell numbers (shown by decreased TUNEL- and cleaved caspase3-positive cells), lower necrotic core area and ratio, and reduced expression of NF-κB target gene. In RAW264.7 cells, XZK inhibited 7-KC-induced upregulation of apoptosis, protein expression of apoptotic markers (cleaved caspase-3 and cleaved PARP), and NF-κB activation (shown by target gene transcription and IκBα reduction). Collectively, our results suggest that XZK effectively suppresses vulnerable plaque progression and rupture by mitigating macrophage ER stress and consequently inhibiting apoptosis and the NF-κB pro-inflammatory pathway, thereby providing an alternative therapeutic strategy for stabilizing atherosclerotic plaques.

## Introduction

Atherosclerotic plaque destabilization and rupture with thrombosis is the central pathologic mechanism responsible for acute vascular events such as acute myocardial infarction and sudden coronary death[1, 2]. Mounting evidence suggests that excessive endoplasmic reticulum (ER) stress plays a significant role in atherosclerotic plaque progression, destabilization, and rupture[3]. The ER is a dynamic and complex organelle in eukaryotic cells responsible for lipid synthesis, Ca2+ storage, and protein folding[4]. ER stress is caused by diverse cellular stresses such as redox imbalance, perturbation of Ca2+ homeostasis, or protein folding defects[4]. To alleviate ER stress, cells activate the unfolded protein response (UPR). The UPR in mammalian cells is mediated by the activation of three ER membrane sensors/transducers: inositol-requiring protein 1 (IRE1), protein kinase RNA-like ER kinase (PERK), and activating transcription factor 6 (ATF6)[5]. Activation of the UPR at appropriate times helps cells to maintain homeostasis and survival. However, prolonged activation of the UPR can lead to apoptosis and an inflammatory response[6, 7], both of which play key roles in plaque instability[8–12]. There is abundant evidence that the UPR is chronically activated in the atherosclerotic lesions of apolipoprotein E knockout (ApoE^-/-^) mice fed chow or Western diet-fed[13, 14], as well as those of human atherosclerotic coronary and carotid arteries, especially thin-cap atheromas and ruptured plaques[15, 16]. ER stress-inducing agents can accelerate atherosclerosis and promote pro-atherosclerotic cellular responses, including cholesterol accumulation, apoptosis, and activation of inflammatory pathways in vascular cells[17–20]. In contrast, the ER stress-alleviating chemical agents phenylbutyrate and tauroursodeoxycholic acid attenuated atherosclerotic lesion progression in mice[21, 22]. Therefore, targeting ER stress may represent a new promising therapeutic strategy for combating plaque instability and rupture.

Cholestin is the fermentation product of rice and red yeast called *Monascus purpureus* and has been regarded as a dietary supplement and traditional medicine with circulation-promoting effects in China and other countries for centuries[23–25]. Xuezhikang (XZK), an extract of Cholestin, has been widely used for the treatment of patients with cardiovascular diseases[23, 26], and has been approved by US Food and Drug Administration (FDA) as a dietary supplement[27–29]. XZK contains 13 natural statins, ergosterol, unsaturated fatty acids, flavonoids, alkaloids, and other biologically active substances[23, 30–32]. Clinical trials have shown that XZK treatment significantly decreases the occurrence of new cardiovascular events, recurrent coronary events, and mortalities[31]. Pharmacological research has demonstrated that treatment with XZK has various therapeutic effects such as lowering lipid levels, suppressing inflammation, and improving endothelial cell function[23, 28]. However, the effects of XZK treatment on plaque vulnerability and their underlying mechanisms are still elusive. In the present study, we used a mouse model of spontaneous vulnerable plaque formation and rupture to investigate whether XZK would improve atherosclerotic plaque stability and clarify the underlying mechanisms. Our results demonstrated that XZK could stabilize atherosclerotic plaques through alleviating ER stress and consequently inhibiting apoptosis and NF-κB pro-inflammatory pathway.

## Materials and methods

### Materials

XZK powder was kindly provided by the WBL Peking University Biotech Co., Luye Pharma Group (Beijing, China). 7-ketocholesterol (7-KC), Oil red O and sirius red were purchased from Sigma-Aldrich (St. Louis, MO, USA). Atorvastatin was obtained from Pfizer Ltd. (New York, NY, USA). The antibodies against α-actin, β-actin, MMP8, MMP13, TNFα, phosphorylated IRE1α (p-IRE1α), IRE1α, eIF2α, PERK and IκBα were from Abcam (Cambridge, MA, USA); antibodies against BiP, phosphorylated PERK (p-PERK), phosphorylated eukaryotic initiation factor 2α (p-eIF2α), spliced x-box binding protein 1 (s-XBP1), cleaved PARP, and active caspase-3 were from Cell Signaling Technology (Beverly, MA, USA); and antibodies against CCAAT-enhancer-binding protein homologous protein (CHOP) and ATF6 were from Santa Cruz Biotechnology Inc. (Santa Cruz, CA, USA). Secondary antibodies including Alexa Fluor 488-labeled donkey anti-rabbit antibody, Alexa Fluor 647-labeled goat anti-rat antibody, and Alexa Fluor 555-labeled donkey anti-mouse antibody were from Invitrogen (Carlsbad, CA, USA). The TUNEL assay kit (In Situ Cell Death Detection kit) was from Roche (Mannheim, Germany), real-time PCR (qPCR) reagent kits were from TAKARA Biotechnology (Dalian, China), and 6-diamino-2-phenylindole (DAPI) was from Beyotime Biotechnology (Shanghai, China).

### Animals and experimental protocol

All animal studies protocols were performed in accordance with the Shanghai Jiaotong University School of Medicine guidelines for the ethical care of animals and approved by the Medical Ethics Committee of Shanghai Jiaotong University. Seven-week-old female ApoE^-/-^ C57BL/6 mice were purchased from Jackson Laboratory (Bar Harbor, ME, USA). At the age of eight weeks, combined partial ligation of the left common carotid artery (LCCA) and left renal artery was carried out under a dissecting microscope as we previously described[33], then the animals were divided into four groups: control (saline, n = 16), atorvastatin (10 mg/kg/d, n = 16), XZK-600 (600 mg/kg/d, n = 19) and XZK-1200 (1200 mg/kg/d, n = 21). Both atorvastatin and XZK were dissolved in isosmotic saline and administered intragastrically for 8 weeks after surgery, and then the mice were euthanized by cervical dislocation and the carotid arteries were collected for various assessments.

### Cell culture

Raw264.7 cells were obtained from the Type Culture Collection of the Chinese Academy of Sciences (Shanghai, China), cultured in DMEM (Thermo Fisher Scientific) supplemented with 10% FBS (Biological Industries, Beit-Haemek, Israel) and antibiotics (100 U/ml penicillin and streptomycin) in a humidified incubator containing 5% CO_2_ at 37°C. Cells were passaged for fewer than 2 months after resuscitation and were used at the third through tenth passage for this study.

### Oil red O and sirius red staining

To assess the collagen and cholesteryl ester content of cells, serial carotid artery cryosections were stained with sirius red and oil red O, respectively, as we previously described[16]. Briefly, to detect collagen in atherosclerotic lesions, frozen sections were incubated in 0.1% sirius red in saturated picric acid for 60 min followed by 1% acetic acid for 30 min, then the slides were mounted with glycerin. The lipid content of the vascular intima was evaluated by oil red O staining. Specimen sections were incubated in a working solution of oil red O for 30 mins, then rinsed in 60% isopropanol for 5 s. The slides were then washed with PBS, counterstained in hematoxylin, differentiated in acid alcohol, rehydrated, and mounted. For both experiments, slides were visualized under a microscope (Leica DM2500, Tokyo, Japan) and images of the entire section were captured at an identical exposure setting for all sections and assessed using image analysis software (ImageJ, National Institutes of Health, Bethesda, MD, USA).

### Immunofluorescence

Immunofluorescence staining was performed as previously described[16, 33]. In brief, a segment of the LCCA that contained plaques was isolated and stored at -80°C. The segments were fixed in 0.01 M paraformaldehyde in cold PBS (pH 7.4) for 10 min and embedded in optimal cutting temperature compound (Sakura Finetechnical, Tokyo, Japan). Serial cross-sections (5-μm) were cut every 200 μm over a 2-mm length of the carotid artery. The frozen sections were permeabilized with 0.2% Triton X-100 for 10 min at room temperature, stained with the appropriate primary antibody, then incubated with secondary antibodies for 1 h, followed by DAPI staining for 8 mins to visualize the nuclei. The images were captured under an LSM 710 confocal laser scanning microscope system (Zeiss, Oberkochen, Germany).

### RNA extraction and quantitative reverse transcription PCR

Total RNA was extracted from control or XZK-treated Raw264.7 cells using trizol (Invitrogen), according to the manufacturer’s instructions. Total RNA (3 μg) was reverse-transcribed into first-strand cDNA by the RevertAid First-Strand cDNA synthesis kit (Fermentas). Quantitative reverse transcription PCR (qRT-PCR) reactions were conducted using SYBR Green dye and the Roche LightCycler® 480 II system. Primers used in the present study were synthesized by Sangon Biotech (Shanghai, China). The sequences were as follow: 5′-TTCTGTCTACTGAACTTCGGGGTGATCGGTCC-3′ (forward primer) and 5′- GTATGAGATAGCAAATCGGCTGACGGTGTGGG-3′ (reverse primer) for TNFα, 5′-CGAAGACTACAGTTCTGCCATT-3′ (forward primer) and 5′- CGAAGACTACAGTTCTGCCATT-3′ (reverse primer) for IL-1α, 5′- CAACCAACAAGTGATATTCTCCATG-3′ (forward primer) and 5′- GATCCACACTCTCCAGCTGCA-3′ (reverse primer) for IL-1β, 5′-ATGGATGCTACCAAACTGGAT-3′ (forward primer) and 5′- ATGGATGCTACCAAACTGGAT-3′ (reverse primer) for IL-6, 5′- ATGGATGCTACCAAACTGGAT-3′ (forward primer) and 5′- CAGTCACCTCTAAGCCAAAGAAA-3′ (reverse primer) form MMP13, 5′- CAGTCACCTCTAAGCCAAAGAAA-3′ (forward primer) and 5′- GCATTAGCTTCAGATTTACGGGT-3′ (reverse primer) for MCP-1, 5′- TGGTGATTTCTTGCTAACCCC-3′ (forward primer) and 5′- TACACTCCAGACGTGAAAAGC-3′ (reverse primer) for MMP8, 5′-AGGTGACAGCATTGCTTCTG-3′ (forward primer) and 5′-AGGTGACAGCATTGCTTCTG-3′ (reverse primer) for β-actin. The resulting values were normalized to β-actin expression.

### Western blot analysis

Western blotting was performed as previously described[34, 35]. The dilutions of the primary antibodies were: anti-ATF6, 1:1,000; anti-p-eIF2α, 1:1,000; anti-CHOP, 1:1,000; anti-s-XBP1, 1:10,000; anti-p-IRE1α, 1:1,000; anti-β-actin, 1:10,000; anti-p-PERK, 1:1,000; anti-cleaved PARP, 1:1,000; anti-IκBα, 1:3,000; and anti-caspase3, 1:1000. The intensities of the protein bands were quantified by Quantity One (Bio-Rad, Richmond, CA, USA) and normalized to β-actin bands.

### Dual-luciferase assay

To measure NF-κB reporter activity, Raw264.7 cells were seeded into 24-well plates. After 24 h, dual-luciferase reporter plasmids were transiently transfected into the cells using Lipofectamine 3000 (Invitrogen), in accordance with the manufacturer’s instructions. 24 hours later, cells were treated with different dose of XZK for 12 h. Cell lysates were prepared by incubating cells in 1× lysis buffer (Luciferase Assay System, Promega, Madison, WI, USA) for 10 mins. Firefly and Renilla luciferase activity levels were measured using the manufacturer’s dual-luciferase assay protocol.

### Terminal deoxynucleotidyl transferase-mediated dUTP nick end-labeling assay

TUNEL staining was performed using the In Situ Cell Death Detection Kit (Roche, Basel, Switzerland), according to the manufacturer’s instructions. Briefly, LCCA sections were fixed in 4% paraformaldehyde for 20 min and permeabilized with 0.1% Triton X-100 in PBS for 2 min on ice. Sections were then incubated with TUNEL reaction mixture for 60 min at 37°C, followed by DAPI staining for 5 min. The images were captured under the LSM 710 confocal laser scanning microscope system (Zeiss).

### Measurement of superoxide anions

LCCAs were harvested and embedded in optimal cutting temperature compound (Sakura Finetechnical, Tokyo, Japan), then cryosectioned into 5-μm-thick sections. Frozen sections were stained with dihydroethidine (DHE; 2 μmol L^-1^) for 20 min at 37°C, followed by nuclear DNA staining using DAPI for 5 min. Fluorescence was imaged with an LSM 710 confocal laser scanning microscope system (Zeiss).

### Blood lipid analysis

Mice were anesthetized using isoflurane inhalation, and blood samples were collected intracardially by syringe. The plasma was separated by centrifugation for 15 min and stored at -80°C until use. Plasma levels of total cholesterol, triglyceride, LDL cholesterol, and HDL cholesterol were measured using a Hitachi 7180 autoanalyzer (Hitachi High-Technologies Corp, Tokyo, Japan) in accordance with the manufacturer’s instructions.

### Statistical analysis

Results are represented as mean ± SEM. Two-tailed unpaired Student’s t-test or Mann-Whitney U test was used for comparisons between two groups where appropriate. Ordinary one-way ANOVA with Tukey's Multiple Comparison post-test or Kruskal-Wallis test with Tunn's Multiple Comparison post-test was used to compare variables among three or more groups, depending on whether the data were parametric or non- parametric. Differences in the classification and occurrence of adverse events were analyzed with χ^2^ tests. *P* < 0.05 was considered significant. Analyses were conducted using GraphPad Prism 6 (GraphPad Software, Inc, San Diego, CA, USA).

## Results

### Administration of XZK attenuates vulnerable plaque progression and rupture in ApoE^-/-^ mice

**Fig 1:**
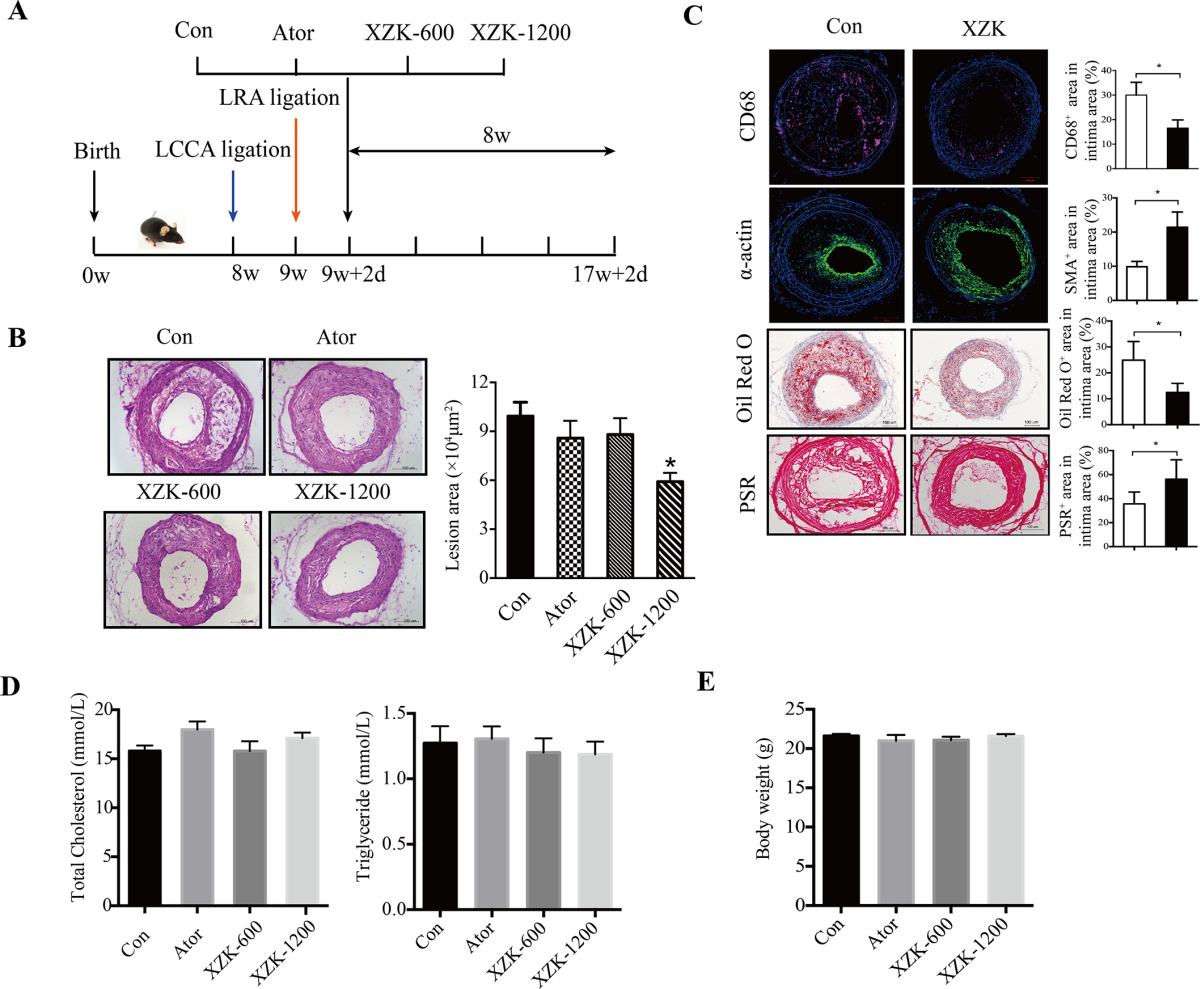
XZK inhibits vulnerable plaque progression and rupture in ApoE-/- mic. (A) The development of a carotid vulnerable plaque in a mouse treated with XZK, atorvastatin, or vehicle. (B) Representative images of H&E staining and quantification of lesion area of carotid arteries at 8 weeks post-ligation in ApoE^-/-^ mice treated with vehicle, atorvastatin (10 mg kg^-1^), XZK-600 (600 mg kg^-1^), or XZK-1200 (1200 mg kg^-1^) once a day by oral administration (n = 16–21 animals per group). (C) Histological analysis of an atherosclerotic lesion in the carotid artery of control and XZK-1200 mice 8 weeks after surgery by staining with CD68, α-smooth muscle actin (α-SMA), oil red O, or sirius red. Quantification of CD68^+^, α-SMA^+^, oil red O^+^, and collagen^+^ areas relative to total lesion area (n = 6 animals per group). (D-E) Total cholesterol and triglyceride levels in plasma (n = 8–13 animals per group) (D) and body weight (n = 19–23 animals per group) (E) of ApoE^-/-^ control, atorvastatin, XZK-600, and XZK-1200 mice 8 weeks after surgery. *P < 0.05 versus control. Abbreviations: LRA, left renal artery; LCCA, left common carotid artery; PSR, PicricSiriusred; Ator, atorvastatin; XZK, Xuezhikang; XZK-600, ApoE^-/-^ mice treated with XZK (600 mg kg^-1^) for 8 weeks after surgery; XZK-1200, ApoE^-/-^ mice treated with XZK (1200 mg kg^-1^) for 8 weeks after surgery.

To investigate the effects of XZK on vulnerable atherosclerotic plaque progression, ApoE^-/-^ mice undergoing combined partial ligation of the left renal artery and left common carotid artery (LCCA) as we previously described[33] were randomly assigned to the following groups: control, XZK-600 (600 mg/kg/d), XZK-1200 (1200 mg/kg/d), and atorvastatin (10 mg/kg/d) as a positive control (Fig 1A). At 8 weeks after surgery, all control group mice had developed atherosclerotic lesions with vulnerable phenotypes in the LCCA. However, only 62.5%, 63.2%, and 52.4% of mice showed vulnerable phenotype lesions in the atorvastatin, XZK-600, and XZK-1200 groups, respectively (*P* < 0.05). Compared to the control group, the treated mice also exhibited a significantly decreased tendency to experience intraplaque hemorrhaging, a reduced incidence of vessel multilayer with discontinuity, and a lower incidence of plaque rupture with thrombus (all comparisons *P* < 0.05 vs. control group; Table 1). These results indicate that XZK treatment protected ApoE^-/-^ mice from vulnerable plaque progression and rupture as effectively as atorvastatin treatment.

**Table 1:** Effects of XZK and atorvastain on lesion features in ApoE-/- mice.

Mice	Stable Phenotype	Vulnerable Phenotype	IntraplaqueHemorrhage	Multilayer With Discontinuity	Rupture With Thrombus
Control (n = 16)	0%(0)	100%(16)	81.25%(13)	81.25%(13)	43.75%(7)
Ator (n = 16)	37.5%(6) *	62.5%(10) *	31.25%(5) *	50%(8) *	12.5%(3) *
XZK-600 (n = 19)	36.8%(7)*	63.2%(12)*	36.8%(7)*	31.6%(6)*	10.5%(2)*
XZK-1200 (n = 21)	47.6%(10)*	52.4%(11)*	33.3%(7)*	33.3%(7)*	9.5%(2)*

Lesion features of ApoE^-/-^ mice treated with vehicle, atorvastatin (10 mg kg^-1^), XZK-600 (600 mg kg^-1^), or XZK-1200 (1200 mg kg^-1^) for 8 weeks after surgery, including the percentage of mice with a stable phenotype, vulnerable phenotype, intraplaque hemorrhage, multilayer plaque with discontinuity, and rupture with thrombus.

* *P* < 0.05 vs group control.

We next examined plaque morphology using histological analysis of serial sections. As depicted in Fig 1B, both the atorvastatin and XZK-600 group mice developed smaller lesions than the control group mice, but this difference was not statistically significant (88052 ± 9980 vs. 99316 ± 8512 μm^2^, *P* > 0.05; Fig 1B). However, the lesions were significantly smaller in XZK-1200 group mice compared with control group mice (59131 ± 5548 vs. 99316 ± 8512 μm^2^, *P* < 0.05; Fig 1B). Moreover, XZK-1200 group mice exhibited significantly smaller CD68-positive areas (23.1 ± 2.6% vs. 37.4 ± 2.6%, *P* < 0.05) and oil red O-positive areas (12.5 ± 1.4% vs. 23.1 ± 3.6%, *P* < 0.05), and larger α-smooth muscle actin (α-SMA)-positive areas (21.4 ± 2.2% vs. 10.7 ± 1.0%, *P* < 0.05) and collagen-positive areas (56.2 ± 6.6% vs. 35.7 ± 4.0%, *P* < 0.05) than control group mice (Fig 1C).

### The effects of XZK are independent of serum lipid levels

We next examined whether XZK and atorvastatin suppressed vulnerable plaque progression and rupture by regulating lipid profiles. Our data showed that no significant differences were observed in serum triglyceride levels [1.27 ± 0.13 mmol/L in control group vs. 1.22 ± 0.78 mmol/L in atorvastatin group (*P* > 0.05), 1.20 ± 0.11 mmol/L in XZK-600 group (*P* > 0.05), and 1.19 ± 0.10 mmol/L in XZK-1200 group (*P* > 0.05)] or total cholesterol levels [15.79 ± 0.57 mmol/L in control group vs. 17.98 ± 0.83 mmol/L in atorvastatin group (*P* > 0.05), 14.54 ± 1.00 mmol/L in XZK-600 group (*P* > 0.05), and 17.10 ± 0.57 mmol/L in XZK-1200 group (*P* > 0.05); Fig 1D]. In addition, there were no significant differences in the levels of LDL and HDL cholesterol in these four groups (S1 Table). Furthermore, the body weight of the mice from the groups was similar after 8 weeks of treatment [21.62 ± 0.24 g in control group vs. 20.98 ± 0.75 g in atorvastatin group (*P* > 0.05), 21.07 ± 0.43 g in XZK-600 group (*P* > 0.05), and 21.60 ± 0.25 g in XZK-1200 group (*P* > 0.05); Fig 1D]. We did not observe any hepatotoxicity or nephrotoxicity upon administration of atorvastatin or XZK at the dose that effectively inhibited vulnerable plaque progression, as evidenced by the absence of a change in expression level of alanine transaminase, aspartate aminotransferase, blood urea nitrogen, and serum creatinine (Scr) in those four groups (S1 Table). Together, these results suggest that the protective effects of XZK on vulnerable plaque progression and rupture are most likely independent of the regulation of serum lipid levels.

### Administration of XZK reduces ER stress in vivo and in vitro

**Fig 2:**
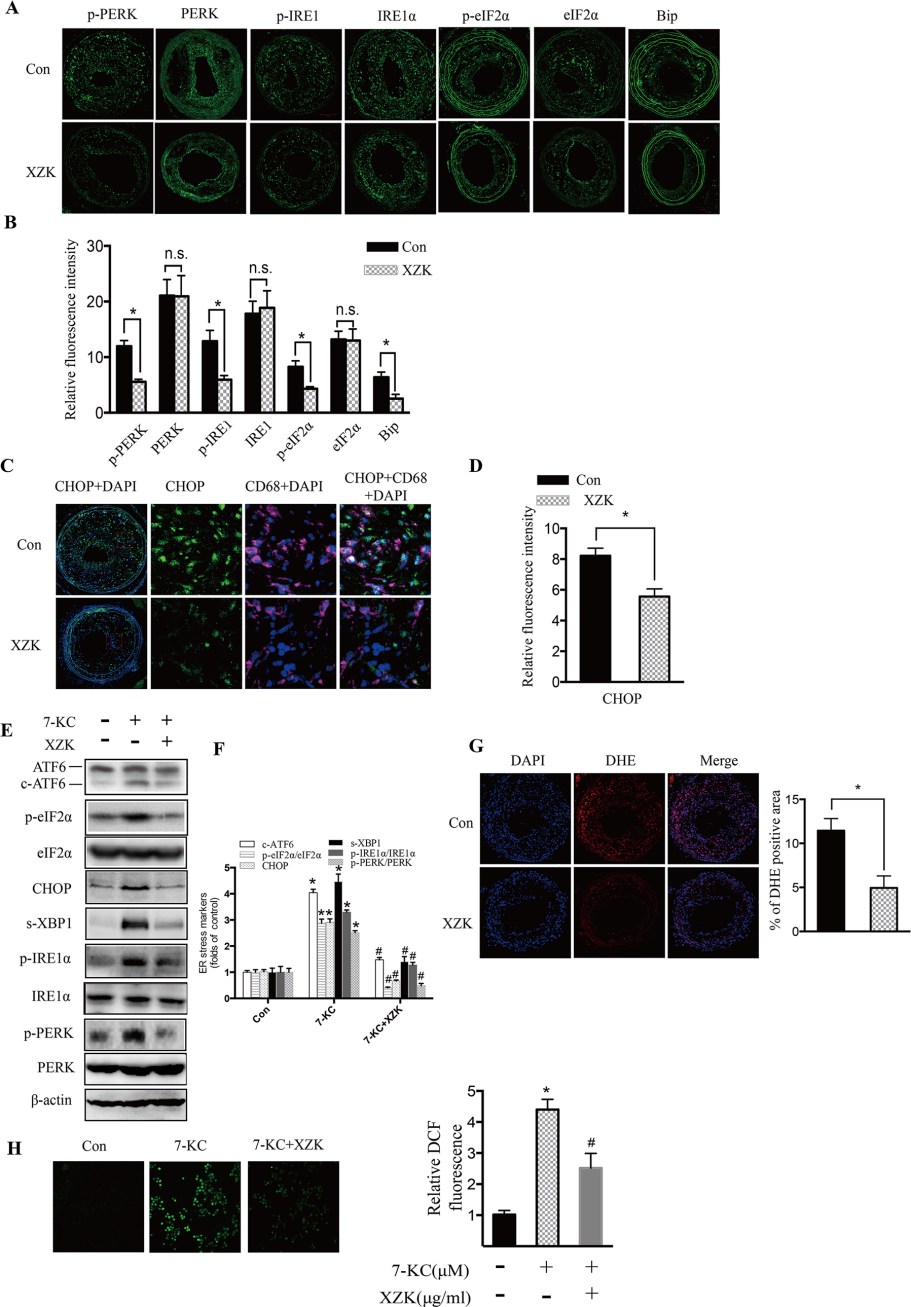
XZK treatment protects against lesional macrophage ER stress. (A-B) Immunofluorescence staining of ER stress markers (p-PERK, p-IRE1, p-eIF2α, and BiP), and total PERK, IRE1α and eIF2α in lesions from ApoE^-/-^ control and XZK-treated mice 8 weeks after surgery (A) and quantification of relative fluorescence intensity (n = 5 animals per group) (B). (C-D) Dual immunofluorescence staining of CD68 and CHOP (C) and quantification of relative fluorescence intensity (n = 5 animals per group) (D). (E-F) RAW264.7 cells pretreated with XZK (100 μg/ml) for 1 h were stimulated by 7-KC (70 μM) for 12 h, and ER stress markers (c-ATF6, p-eIF2α, CHOP, s-XBP-1, p-IRE1α, and p-PERK) and total eIF2α, IRE1α and PERK were analyzed by western blot (E) and subjected to semi-quantitative analysis (n = 3) (F). (G) DHE staining of lesions from ApoE^-/-^ control and XZK-treated mice 8 weeks after surgery and quantification of relative fluorescence intensity (n = 5 animals per group). (H) RAW264.7 cells pretreated with XZK (100 μg/ml) for 1 h were stimulated by 7-KC (70 μM) for 12 h, and DCF fluorescence formation was visualized and quantified (n = 6). *P < 0.05 versus control; ^#^*P* < 0.05 versus 7-KC-treated along. Abbreviations: XZK, Xuezhikang; DAPI, 4’,6-diamidino-2-phenylindole; 7-KC, 7-ketocholesterol; DHE, Dihydroethidium; DCF, Dichlorofluorescein.

Mounting evidence has demonstrated that prolonged and severe ER stress is a key contributor to plaque vulnerability and acute cardiac death[6, 36]. To elucidate whether XZK could mitigate ER stress *in vivo*, we examined the expression of ER stress indicators in serial sections of carotid arteries from high-dose XZK-treated and control ApoE^-/-^ mice. Immunofluorescence analysis showed a significant reduction in the expression of p-PERK, p-IRE1α, p-eIF2α, and BiP in the lesions of the XZK-treated group when compared with the control group (*P* < 0.05; Fig 2A and 2B). No significant difference was observed in the expression levels of total PERK, IRE1α and eIF2α in the control and XZK-treated groups (Fig 2A and 2B). The macrophage CCAAT-enhancer-binding protein homologous protein (CHOP) expression levels were also decreased in the XZK-treated group (*P* < 0.05; Fig 2C and 2D). We further investigated the regulatory effect of XZK on ER stress in RAW264.7 macrophages. As shown in Fig 2E and 2F, pretreatment with XZK mitigated ER stress induced by 7-KC, an atherosclerotic lesional oxysterol that promotes oxidative stress and ER stress in macrophages, demonstrated by the reduced expression of cleaved-ATF6 (c-ATF6), CHOP and s-XBP1, and the decreased phosphorylation of eIF2α, inositol-requiring enzyme 1α (IRE1α), and PERK. Together, these findings indicate that administration of XZK effectively suppressed the ER stress response *in vivo* and *in vitro*.

Previous studies have shown that elevation of intracellular ROS is the universal mechanisms for aberrant ER stress and UPR activation[37, 38]. Therefore, we assessed whether administration of XZK suppressed ER stress by altering intracellular ROS levels. As shown in Fig 2G, the intensity of dihydroethidine (DHE)-derived fluorescence in carotid aortic sections of XZK-treated mice was significantly decreased compared with that of control mice. Consistent with this result, RAW264.7 cells stimulated with 7-KC showed a drastically increased level of ROS, and XZK treatment dramatically reduced these levels (Fig 2H). Thus, these results suggest that the inhibitory effects of XZK on ER stress are most likely mediated by the regulation of intracellular ROS levels.

### XZK attenuates macrophage apoptosis and necrotic core formation

**Fig 3:**
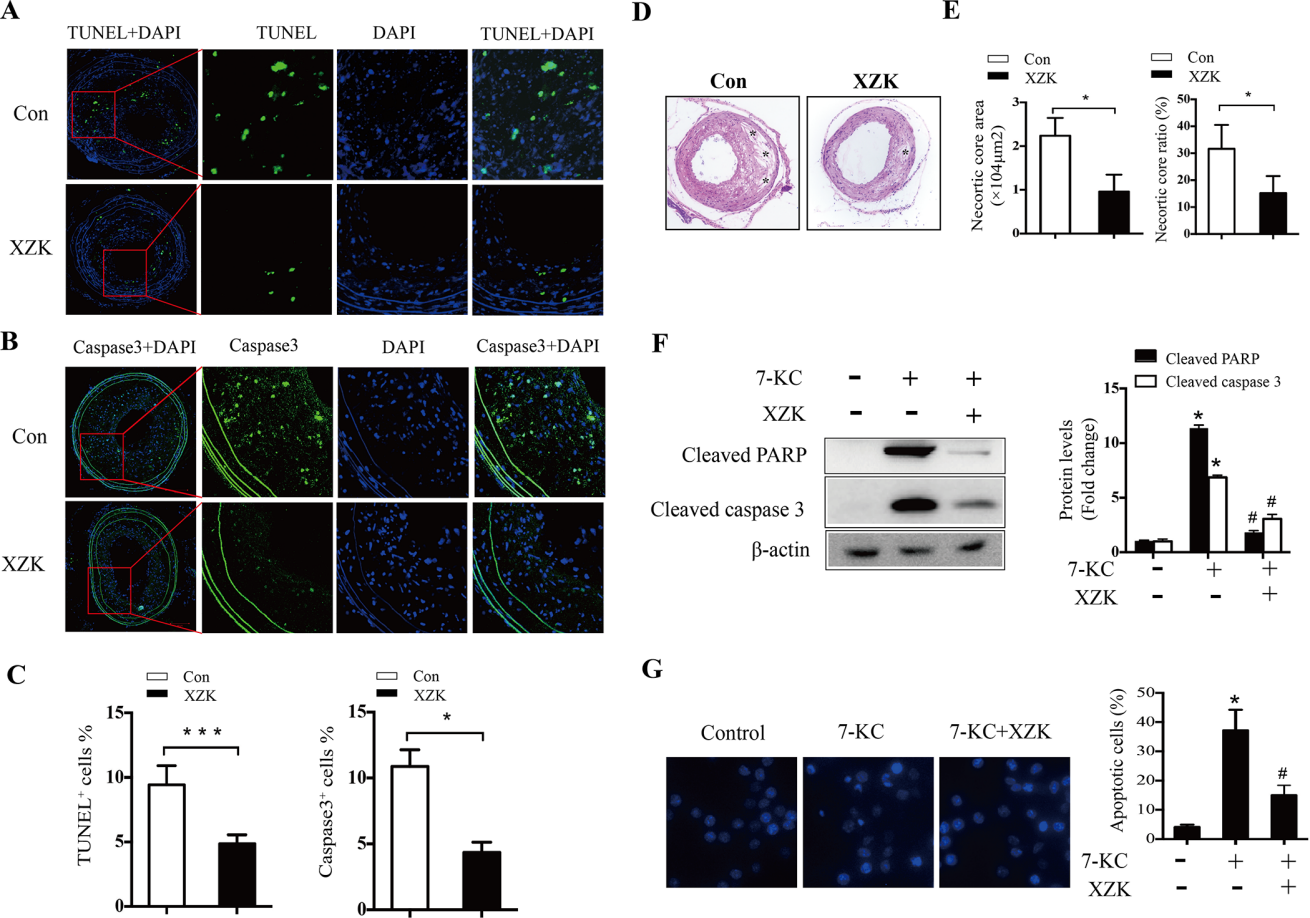
Effect of XZK on macrophage apoptosis. (A-B) Representative images of apoptotic cells in lesions from ApoE^-/-^ control and XZK-treated mice as determined by TUNEL assay (A) and immunofluorescence staining of cleaved caspase-3 (B). (C) Quantification of TUNEL^+^ (left) and cleaved caspase-3^+^ (right) cells (n = 4–5 animals per group). (D-E) H&E staining in lesions from ApoE^-/-^ control and XZK-treated mice (*, necrotic areas) (D) and quantification of necrotic core area (E, left) and ratio (E, right) (n = 5 animals per group). (F) RAW264.7 cells were treated with 7-KC (70 μM) in the absence or presence of XZK (100 μg/ml) for 16 h. PARP and caspase-3 cleavage were analyzed by immunoblotting (n = 3). (G) RAW264.7 cells were treated with 7-KC (70 μM) in the absence or presence of XZK (100 μg/ml) for 16 h. Nuclear morphology was examined by DAPI staining (left) and the number of apoptotic cells was quantitated (n = 5). *P < 0.05 or ***P < 0.001 versus control; ^#^*P* < 0.05 versus 7-KC-treated along. Abbreviations: XZK, Xuezhikang; DAPI, 4’,6-diamidino-2-phenylindole; 7-KC, 7-ketocholesterol.

ER stress-induced apoptosis is strongly linked with the expansion of the necrotic core (NC) and the progression and rupture of vulnerable plaques, both in mouse lesions and in human coronary and carotid arteries[15, 16]. Therefore, we evaluated the anti-apoptotic effects of XZK by measuring the proportion of apoptotic cells using TUNEL staining. Our results showed a significant reduction in the percentage of TUNEL-positive cells in the lesions from XZK-treated mice compared with control group mice [4.8 ± 0.28% vs. 9.24 ± 0.6%, *P* < 0.05; Fig 3A and 3C (left)]. Furthermore, as seen in Fig 3B, XZK dramatically decreased the percentage of cells in lesions positive for cleaved caspase-3 (a key executor of apoptosis) [4.4 ± 0.4% vs. 10.9 ± 0.6%, *P* < 0.05; Fig 3B and 3C (right)]. Moreover, the atherosclerotic plaques in XZK-treated mice contained smaller NC areas and ratios compared with control mice (NC area: 7699 ± 2440 vs. 22381 ± 2037 μm^2^, *P* < 0.05; NC ratio: 12.1 ± 3.9% vs. 31.6 ± 5.1%, *P* < 0.05; Fig 3D and 3E). We further determined the anti-apoptotic effects of XZK in RAW264.7 macrophages. As shown in Fig 3F and 3G, XZK pretreatment significantly inhibited 7-KC-induced macrophage apoptosis, as evidenced by the reduced expression of cleaved PARP and cleaved caspase-3 (Fig 3F), and decreased percentage of cells with nuclear fragments (Fig 3G). Taken together, these results suggest that administration of XZK protects against macrophage apoptosis and NC expansion.

### XZK inhibits NF-κB pro-inflammatory signaling pathway

**Fig 4:**
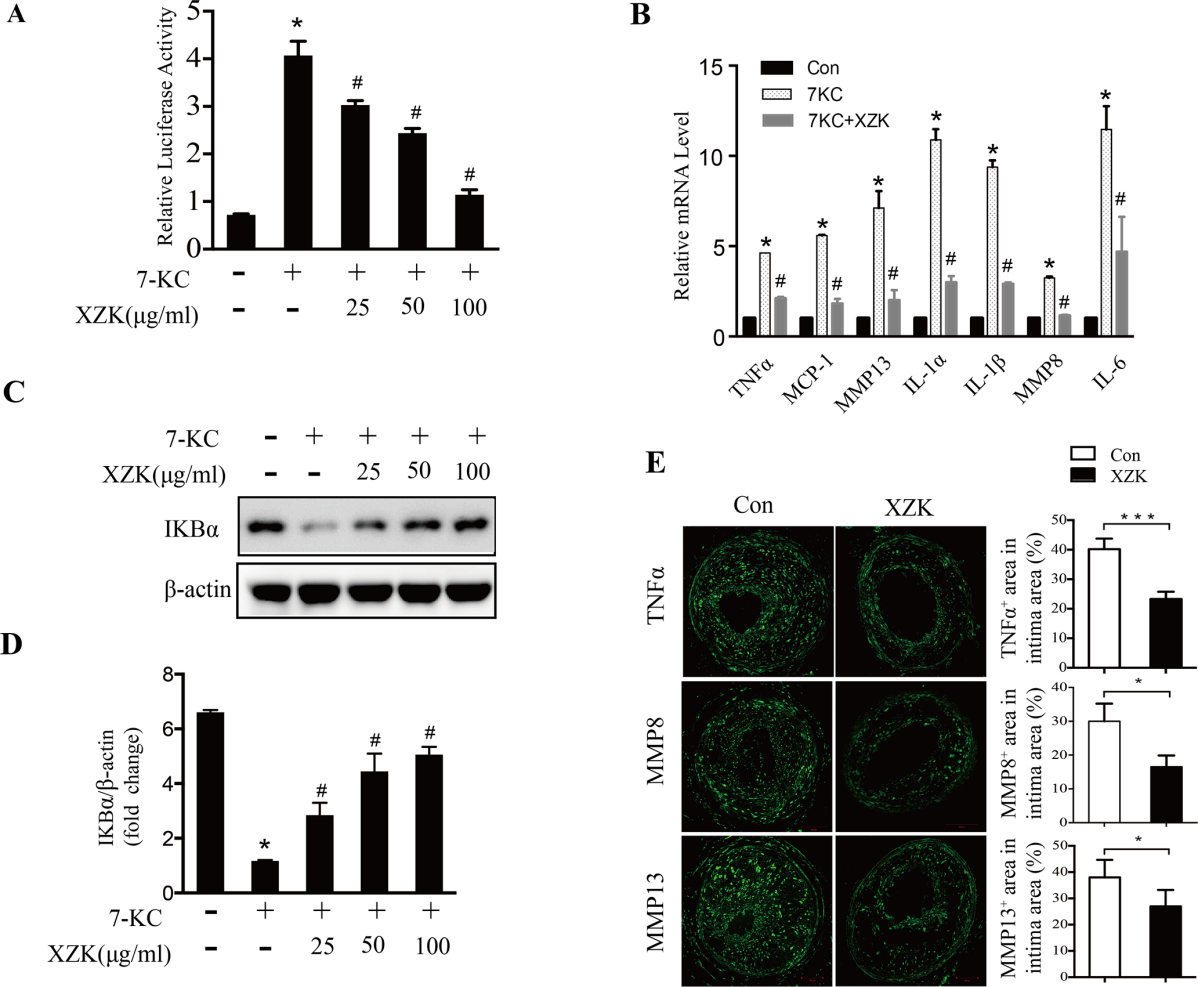
XZK mitigates NF-κB inflammatory pathway. (A) RAW264.7 cells transfected with NF-κB-luciferase reporter and renilla luciferase were treated with 7-KC (70 μM) in the presence or absence of the indicated concentration of XZK for 12 h. Luciferase activities were measured, and renilla luciferase activity normalized to firefly luciferase activity and plotted as relative luciferase activity (n = 5). (B-D) RAW264.7 cells were treated with 7-KC (70 μM) alone or together with XZK (25, 50 and 100μg/ml) for 12 h. The mRNA levels of the inflammatory markers (mean ± SEM., n≥3) were measured by qRT-PCR (B). IκBα protein levels were analyzed by immunoblotting (n = 3) (C) and subjected to semi-quantitative analysis (n = 3) (D). (E) Representative images of immunofluorescence staining of TNFα, MMP8, and MMP13 in plaques from ApoE-/- control and XZK mice 8 weeks after surgery, and quantification of their positive areas (n = 5 animals per group). *P < 0.05 or ***P < 0.001 versus control; ^#^*P* < 0.05 versus 7-KC-treated along. Abbreviations: XZK, Xuezhikang; 7-KC, 7-ketocholesterol.

In addition to the induction of apoptosis, prolonged ER stress can also activate pro-inflammatory reactions, which are mainly mediated by the transcription factor NF-κB. Therefore, we determined the effect of XZK on the NF-κB pro-inflammatory signaling pathway in macrophages. Our reporter gene assay data showed that administration of XZK could significantly inhibit 7-KC-mediated NF-κB activation in a dose-dependent manner (Fig 4A). Consistently, 7-KC-induced transcription of NF-κB target genes, including *TNFα*, *MCP-1*, *MMP13*, *IL-1α*, *IL-1β*, *MMP8*, and *IL-6*, was inhibited by XZK (Fig 4B). Further analysis revealed that XZK prominently inhibited the 7-KC-induced reduction of IκBα levels (Fig 4C and 4D). Moreover, we analyzed the expression of inflammatory cytokines in carotid lesions in the control and XZK-treated mice. Immunostaining data showed that the expression of *TNFα*, *MMP8*, and *MMP13* were significantly lower in the XZK group than in the control group (Fig 4E). Taken together, these data suggest that XZK could effectively suppress the NF-κB pro-inflammatory pathway *in vitro* and *in vivo*.

## Discussion

Atherosclerotic plaque rupture with thrombosis is the leading cause of acute cardiovascular events, and therapies aimed at stabilizing vulnerable atherosclerotic plaques are of great clinical significance. However, effective intervention strategies for stabilizing vulnerable plaques remain largely limited. A large body of clinical and animal studies has demonstrated the athero-protective effects of XZK. However, whether XZK can suppress vulnerable atherosclerotic plaque progression and rupture is still unclear. A significant finding presented here is that chronic oral administration of XZK prominently suppressed atherosclerotic vulnerable plaque progression and rupture in our previously developed mouse model of spontaneous vulnerable plaque formation and rupture in carotid arteries in ApoE^-/-^ mice, thus providing an alternative therapeutic strategy for suppressing vulnerable plaque progression and rupture.

Mounting evidence derived from human atherosclerotic lesions and mouse models of atherosclerosis demonstrated that ER stress plays a major role in vulnerable plaque progression and rupture[3, 13, 14]. In the present study, we found that XZK treatment in mice significantly alleviated ER stress in atherosclerotic lesions, as evidenced by the decreased expression of ER stress markers such as p-PERK, p-IRE1α, p-eIF2α, and BiP in treated mice relative to controls. Furthermore, 7-KC-induced expression of ER stress markers was also inhibited by XZK treatment in RAW264.7 macrophages. These results indicate that XZK might suppress vulnerable plaque progression through the mitigation of ER stress.

Macrophage apoptosis plays opposing roles in plaque progression. In early lesions, macrophage apoptosis, accompanied by rapid phagocytic clearance of dead cells (i.e., efferocytosis) by neighboring phagocytes, suppresses plaque progression. In advanced lesions, however, macrophage apoptosis, coupled with defective efferocytosis, promotes the expansion of the lipid core and consequently results in necrosis, inflammation, and even plaque rupture[8, 9]. Increasing evidence has demonstrated that prolonged and severe ER stress plays a key role in advanced lesional macrophage apoptosis, and this is mainly mediated by CHOP, a specific pro-apoptotic protein under condition of ER stress. CHOP expression is upregulated in advanced lesions and contributes to the instability of atherosclerotic plaques, while CHOP deficiency in advanced atherosclerotic lesions decreases apoptosis and plaque necrosis[39, 40]. In this study, we observed that XZK could attenuate 7-KC-induced CHOP upregulation, PARP and caspase-3 cleavage, and macrophage apoptosis in RAW264.7 cells. Furthermore, the amount of apoptosis, NC area and ratio, and CHOP expression levels were decreased in atherosclerotic lesions of XZK-treated ApoE^-/-^ mice. The phosphorylation of PERK plays a dominant role in the ER stress-CHOP apoptotic pathway[41]. Here, we found that XZK significantly reduced the expression of c-ATF6 and phosphorylation of PERK and eIF2α in 7-KC-treated macrophages. Together, these results indicate that XZK may suppress the activation of two critical upstream signals, cleavage of ATF6 and phosphorylation of PERK, and consequently inhibit the ER stress-CHOP apoptotic pathway.

Apart from triggering apoptosis, a chronic ER stress response also induces inflammation, which is widely accepted to play a key role in determining plaque instability. NF-κB, which is mainly activated by the IRE1 and PERK pathways under ER stress conditions, is a central mediator of ER stress-induced pro-inflammatory pathways[42]. Phosphorylated IRE1α and PERK activate NF-κB by promoting IκBα degradation and inhibiting IκBα transcription, respectively. In the present study, we show that XZK inhibited 7-KC-induced phosphorylation of IRE1α and PERK, activation of NF-κB, reduction of IκBα protein levels, and increased transcription of NF-κB target genes in RAW264.7 macrophages. In addition, the expression of phosphorylated IRE1α, PERK, and NF-κB targeting genes, such as TNFα, MMP8, and MMP13 were reduced in the atherosclerotic lesions of XZK-treated ApoE^-/-^ mice. These data suggest that XZK may mitigate the NF-κB pro-inflammatory pathway via suppression of the ER stress-triggered IRE1α and PERK pathway.

It is well known that statin’s lipid-lowering and pleiotropic effects are dose dependent. High-dose atorvastatin decreased plasma cholesterol and inhibited atherosclerosis development. In our previous study, we found low dose of atorvastatin (10mg/kg/day) improved plaque stability with minimal effects on atherosclerotic plaque progression, and did not affect serum lipids levels[43]. The purpose of the present study was to investigate whether vulnerable plaques in our mouse model would respond to XZK treatment independent of plasma cholesterol levels. So in present study, we choose 10mg/kg/day atorvastatin as well as corresponding dose of XZK. Our results showed that oral administration of XZK could remarkably decrease the percentage of vulnerable phenotype lesions and incidence of plaque rupture with thrombus, as well as the incidence of intraplaque hemorrhage and discontinuity of multilayer vessels as effectively as atorvastatin treatment. Meanwhile, we did not detect any significant differences in the levels of total serum cholesterol, triglycerides, LDL cholesterol, and HDL cholesterol among atorvastatin, XZK-600, XZK-1200, and control mice in our system, indicating that XZK can exert its athero-protective effect in a manner independent of lipid lowering.

Elevation of ROS is generally considered to be a major mechanism of cellular ER stress[44]. Here, we found significantly reduced DHE-derived fluorescence in the atherosclerotic lesions of XZK-treated mice. In addition, XZK strongly suppressed the 7-KC-induced upregulation of 2',7'-dichlorofluorescein (DCF) fluorescence in RAW264.7 macrophages, suggesting that XZK may alleviate ER stress by decreasing ROS accumulation. ROS can be generated from a variety of sources in atherosclerotic lesions. One of the major sources of ROS is the mitochondria. In addition, several extramitochondrial enzymes, including NADPH oxidases, uncoupled nitric oxide synthase, xanthine oxidase[45], cyclooxygenase[46], myeloperoxidase[47], and lipoxygenase[48] contribute to ROS accumulation. Thus, the mechanism by which XZK inhibits ROS accumulation should be further investigated. Aside from increased ROS, decreased Ca^2+^ concentration in the ER lumen is another mechanism leading to aberrant ER stress and UPR activation[44, 49]. The ER is the major intracellular Ca^2+^ store, containing about 2 mM total Ca^2+^, which is roughly fourfold higher than the cytoplasmic free Ca^2+^ concentration[44]. A decrease in ER Ca^2+^ concentration results in elevation of cytoplasmic calcium. In this study, we found that RAW264.7 cells stimulated with 7-KC showed increased intracellular Ca^2+^ concentration, while XZK treatment dramatically reduce these levels (S1 Fig), indicating that XZK may also alleviate ER stress through the regulation of ER Ca^2+^ levels. However, further experiments should be conducted to confirm this hypothesis.

In conclusion, XZK can inhibit vulnerable plaque progression and rupture in ApoE^-/-^ mice in a manner independent of lipid regulation. Rather, the beneficial effect of XZK appears to be achieved by suppression of ER stress-mediated apoptosis and the NF-κB pro-inflammatory pathway, thereby providing an alternative strategy to stabilize atherosclerotic plaques.

## Supporting information

S1 Fig Administration of XZK decreases intracellular Ca^2+^ levels. RAW264.7 cells pretreated with the indicated concentration of XZK for 1 hour were stimulated by 7-KC (70μM) for 12h, and the intracellular Ca^2+^ concentration was measured using a Fluo-4 NW kit (n = 5). *P < 0.05 versus control; #P < 0.05 versus 7-KC-treated along (unpaired Student’s t-test). Data are representative of 3 independent experiments. Values are presented as mean ± SEM., n ≥ 4). Abbreviations: XZK, Xuezhikang; 7-KC, 7-ketocholesterol.
(TIF)

S1 Table The effects of XZK on lipid profiles, liver function and renal function of ApoE^-/-^ mice. At the end of study, blood was collected in heparinized tubes from anesthetized mice by left ventricular puncture. Plasma was obtained by centrifugation (5,000 rpm) at 4°C for 10 min and stored at -80°C. Plasma concentrations of LDL cholesterol, HDL cholesterol, ALT, AST, BUN and Scr were measured by appropriate methods. Data represents the mean ± SEM., n ≥ 6.
(TIF)
