# Supplementary figures and images for: Xuezhikang, an extract from red yeast rice, attenuates vulnerable plaque progression by suppressing endoplasmic reticulum stress-mediated apoptosis and inflammation
Source: PLoS One. 2017 Nov 30;12(11):e0188841. doi: 10.1371/journal.pone.0188841 (PMC5708751; doi:10.1371/journal.pone.0188841)

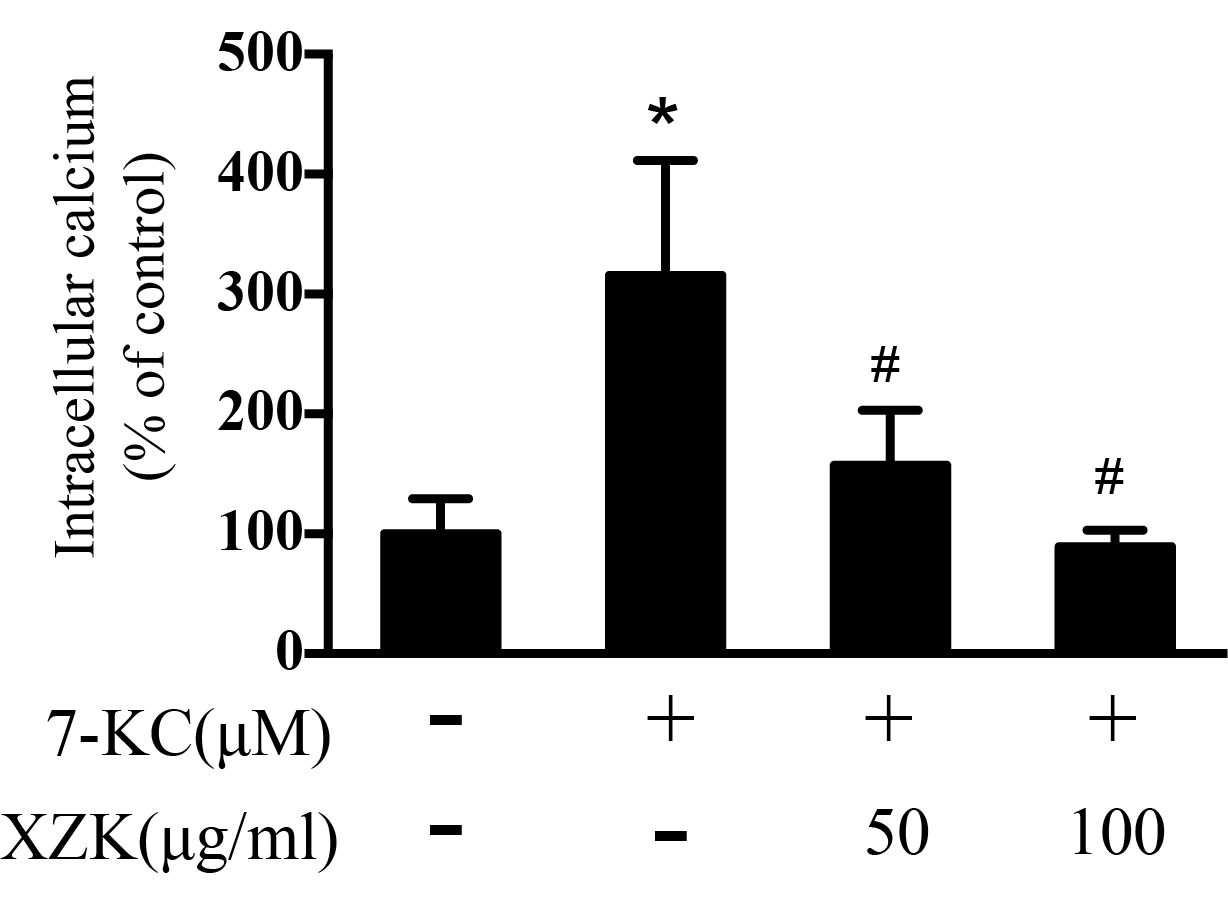

Supplement: S1 Fig — RAW264.7 cells pretreated with the indicated concentration of XZK for 1 hour were stimulated by 7-KC (70μM) for 12h, and the intracellular Ca2+ concentration was measured using a Fluo-4 NW kit (n = 5). *P < 0.05 versus control; #P < 0.05 versus 7-KC-treated along (unpaired Student’s t-test). Data are representative of 3 independent experiments. Values are presented as mean ± SEM., n ≥ 4). Abbreviations: XZK, Xuezhikang; 7-KC, 7-ketocholesterol. (TIF) [file pone.0188841.s001.tif]

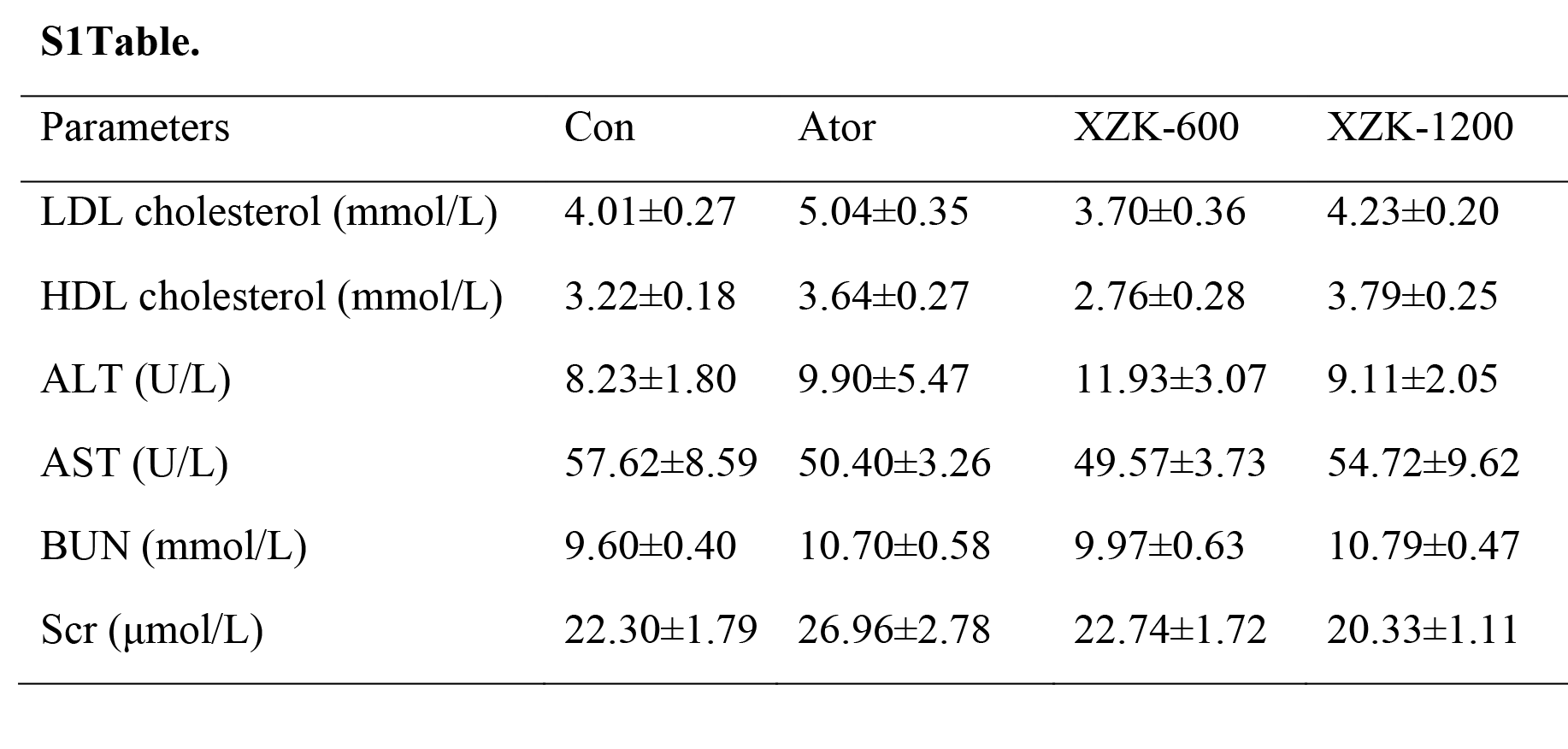

Supplement: S1 Table — At the end of study, blood was collected in heparinized tubes from anesthetized mice by left ventricular puncture. Plasma was obtained by centrifugation (5,000 rpm) at 4°C for 10 min and stored at -80°C. Plasma concentrations of LDL cholesterol, HDL cholesterol, ALT, AST, BUN and Scr were measured by appropriate methods. Data represents the mean ± SEM., n ≥ 6. (TIF) [file pone.0188841.s002.tif]
